# Characteristics of Epoxy Composites Containing Carbon Nanotubes/Graphene Mixtures

**DOI:** 10.3390/polym15061476

**Published:** 2023-03-16

**Authors:** Tatiana P. Dyachkova, Yulian A. Khan, Elena A. Burakova, Evgeny V. Galunin, Gulnara N. Shigabaeva, Dmitry N. Stolbov, Georgy A. Titov, Nikolay A. Chapaksov, Alexey G. Tkachev

**Affiliations:** 1 Research Department «Technology and Methods of Nanoproducts Manufacturing», Technological Institute, Tambov State Technical University, 106/5, Building 2, Sovetskaya St., 392000 Tambov, Russia; 2 Department of Organic and Ecological Chemistry, Institute of Chemistry, University of Tyumen, 6 Volodarskogo St., 625003 Tyumen, Russia; 3 Laboratory of Catalysis and Gas Electrochemistry, Faculty of Chemistry, Lomonosov Moscow State University, 1 Leninskiye Gory, 119991 Moscow, Russia

**Keywords:** carbon nanotubes, graphene nanoplates, graphene oxide, epoxy composites, mechanical properties

## Abstract

The paper considers the development of fillers representing mixtures of carbon nanotubes and graphene materials (graphene oxide and graphene nanoplatelets) in different mass ratios to modify epoxy resin. The graphene type and content effect on the dispersed phase particle effective sizes—both in aqueous suspensions and the resin—was analyzed. Hybrid particles were characterized by Raman spectroscopy and electron microscopy. The composites containing 0.15–1.00 wt.% CNTs/GO and CNTs/GNPs were thermogravimetrically analyzed, and their mechanical characteristics were determined. SEM images of the composite fracture surfaces were acquired. Optimal dispersions containing 75–100 nm particles were obtained at the CNTs:GO mass ratio of 1:4. It was shown that the CNTs can be located between the GO layers and on the GNP surface. The samples containing up to 0.2 wt.% CNTs/GO (at 1:1 and 1:4 ratios) were stable when heated in air up to 300 °C. For 0.15–0.20 wt.% CNTs/GO (at 1:1 ratio), the tensile strength and modulus of the composite increased by 84–88 and 40%, respectively. The increase in the strength characteristics was found to occur due to the interaction of the filler layered structure with the polymer matrix. The obtained composites can be used as structural materials in different fields of engineering.

## 1. Introduction

Carbon nanomaterials (CNMs) are often proposed for using as fillers in advanced polymer composites to improve mechanical properties, as well as thermal stability, thermal and electrical conductivity [1]. The most typical CNMs are carbon nanotubes (CNTs), graphene nanoplatelets (GNPs) and graphene oxide (GO), which represent 1D and 2D graphene-based structures [2] with different geometric parameters and number of layers.

Recently, more and more publications have appeared mentioning the synergistic effect of different types of CNM mixtures on the characteristics of polymer composites. CNTs/graphene mixtures have been used to modify bitumen [3], polypropylene [4], polyimide [5], polycarbonate/ethylene methyl acrylate [6], polyurethane [7], paraffin [8], poly(ether sulfone) [9], poly(vinyl alcohol) [10], poly(vinylidene fluoride) [11], ultrahigh-molecular-weight polyethylene [12], styrene–butadiene rubber [13], etc. The studies show that the addition of graphene improves CNT distribution in the polymer matrix [4,5,8,10], reduces the percolation threshold [4,7], and also contributes to a number of other interesting effects. By changing the CNTs:graphene mass ratio introduced into polymer matrices, it is possible to control their conductivity, percolation thresholds, and other characteristics [14].

For instance, it is shown that GO/CNTs/polyimide nanocomposites exhibit a much lower friction coefficient compared to that of composites with the same GO or CNT contents [5]. The introduction of a CNTs/graphene hybrid filler into polycarbonate/ethylene methyl acrylate in a 1:3 ratio results in the electromagnetic radiation shielding efficiency of up to −34 dB in the frequency range of 8.2–12.4 GHz (X-band) [6].

In [15], it is shown that multiwalled CNTs (MWCNTs) act supplementally to GNPs by forming conductive networks in ethylene–propylene–diene monomer rubber. As a result, at a total CNTs/GNPs filler content of 26.7 vol.%, the tensile strength, Young’s modulus and tear strength of the composites were found to increase by 707, 825 and 428%, respectively, in comparison with the increases of 404, 710 and 270%, respectively, obtained for the composites wherein only GNPs were used. This confirms the synergy between the GNPs and CNTs, since both are carbon-based and have supplementary geometries.

Special attention should be paid to using CNMs in epoxy composites [16,17,18,19]. In the review [20], it is noted that although CNTs and graphene possess ultra-high Young’s modulus and strength, nanoscale size and high specific surface area, the mechanical properties of composites reinforced by them does not appear to be as large as expected. The main reason for this low efficiency is the tendency of nanotubes and graphene to agglomerate in the epoxy resin (ER) matrix. To solve this problem, the CNT and GNP surface is usually functionalized [21,22].

Quite complicated techniques are most often used in this case. In [23], pre-oxidized CNTs were treated with a mixture of SOCl_2_ and N,N-dimethylformamide, after which they were combined with pre-synthesized polysilicon. The CNTs functionalized through this method were injected into an ER to obtain a composite with increased flame resistance. In [24], CNTs oxidized with a mixture of sulfuric and nitric acids (3:1) were modified with Fe_3_O_4_ particles and used to prepare epoxy composites with ordered MWCNTs.

In [25], carbon nanofibers (CNFs) were oxidized in AgNO_3_/K_2_S_2_O_8_ solution at 343 K for 1 h to introduce carboxyl groups, and then were treated with isophorone diisocyanate and imidazole. As a result, a layer of hyperbranched polyimidazole was formed on the CNF surface. The application of the CNFs modified according to this technique made it possible to improve markedly the interfacial shear, interlaminar shear and impact strength of the epoxy composite.

However, one way or another, the formation of the functional groups increases the defectiveness of the nanocarbon filler structure, which does not always have a positive effect on the mechanical and electrophysical properties of composites. Therefore, the shape and number of the graphene layers in the carbon nanomaterial should be considered in any option of chemical treatment, and the degree of functionalization should be controlled [26].

GNPs and GO initially contain a fairly large number of polar groups and can act as stabilizers of fiber material dispersions in polymer matrices. For instance, in [27], the GO was used as a modifying additive for polymer fiber-reinforced composites based on ER and basalt roving. Due to the availability of functional groups on the GO surface, the epoxy hardening (curing) was initiated. The composite formed possessed an increased glass transition temperature, and was thermally stable at 200–350 °C.

GO and GNPs also enhance the CNT properties in epoxy composites. In [17], it is shown that a CNTs/GNPs mixture increases the thermal and electrical conductivity of an epoxy composite to a much greater extent than single CNTs and GNPs.

The work [28] presents a strategy to reinforce/improve simultaneously the mechanical/functional behavior of an epoxy composite. To achieve this, the hybrid filler was modified by Triton-X, which was able to introduce steric repulsive forces between the epoxy matrix and carbon fillers, thereby improving their dispersibility and preserving the internal conductivity of the carbon particles.

The other works also demonstrate interesting effects achieved by the combined use of CNTs and graphene in epoxy composites. In [16], the tensile modulus, tensile strength and thermal conductivity of epoxy composites containing 0.9 wt.% GNPs and 0.1 wt.% CNTs were improved by 22.6, 14.5 and 46.9%, respectively, compared to the native ER. In [18], the mechanical characteristics of CNTs/GO/epoxy resin composites were greatly enhanced by adding 0.04 wt.% CNTs and 0.2 wt.% GO. Approximately a 950% improvement in fatigue life and 400% improvement in creep rupture life were observed at the applied stress levels tested. In [19], enhanced tensile and electrical properties in a GNPs/MWCNTs/epoxy hybrid were achieved at the GNPs:MWCNTs ratio of 0.1:0.4.

Thus, to the best of our knowledge, the information on the optimum composition of hybrid fillers is quite contradictory. Most often, the authors of the cited works do not conduct a systematic study, limiting themselves to a simple statement of the synergistic effect. None of the works has analyzed the graphene component type effect on the properties of the formed hybrid filler. In [29], it is shown that the GNP particle size has a pronounced effect on the mechanical properties and thermal conductivity of epoxy composites. The larger flakes succeed in better reinforcement of the composites. In mixture samples, at the highest CNTs:GNPs ratio (9:1), a marked improvement of 76% in the fracture toughness was observed. However, in addition to the cross-sectional dimensions, graphene materials differ in the number of layers and content of oxygen-containing groups. These characteristics play an important role in the CNT deagglomeration and in formation of bonds with epoxy macromolecules. Besides, earlier researchers did not pay attention to the fact that the CNTs/graphene-based hybrid filler can be formed under different conditions, which can also further affect the composite properties.

Therefore, in the present work, various types of graphene materials (GNPs and GO) were combined with CNTs to obtain hybrid fillers. The graphene material-type effect on the stabilization of aqueous hybrid particles dispersions was evaluated. Besides, the effect of the way of drying and grinding of hybrid particles on their structure and morphology was studied. The dispersion efficiency of experimental nanomodifier samples in the epoxy matrix was analyzed, and the electrical conductivity, thermal stability and mechanical properties of the composites obtained were determined.

## 2. Materials and Methods

### 2.1. Reagents and Materials

MWCNTs, GNPs and GO acquired from NanoTechCenter Ltd (Tambov, Russia) were used in this work. The CNTs were synthesized through the CVD method from a propane-butane mixture with a Co/Mo/Mg/Al catalyst at 630 °C [30]. The BET specific surface area of this nanomaterial is 260 m^2^/g. The impurity content of the metal oxide catalyst does not exceed 1 wt.%.

The GNPs were manufactured through oxidative intercalation of expanded graphite with subsequent ultrasonic treatment. The material contains ~10 wt.% oxygen and <1 wt.% sulfur.

The GO was obtained through the modified Hummers-Offeman’s method [31] in the form of an aqueous suspension containing 1 wt.% of the product. According to the manufacturer’s data, the dry material contains 40 wt.% of oxygen and 2 wt.% of sulfur.

An ED-22 ER (Novokhim Ltd, Naberezhnye Chelny, Russia) based on bisphenol-A type diglycidyl ether was chosen as a polymer binder, and 2,4,6-Tris[(dimethylamino)methyl]phenol [UP-606/2] (Chimex Ltd, St. Petersburg, Russia) was employed as a hardener (curing agent).

### 2.2. Preparing CNTs/GO and CNTs/GNPs Hybrid Particles

To obtain the CNTs/GO filler, the CNTs were mixed with a 1 wt.% GO aqueous suspension so that the CNTs:GO mass ratios were 1:1 and 1:4 (regarding dry materials). The resulting mixture was subjected to ultrasonic treatment for 30 min, after which it was dried under different conditions:

(1) in a desiccator at 40 °C,

(2) in a Scientz-10N freeze dryer (Ningbo Scientz Biotechnology Co, Ningbo, China), where the aqueous paste is frozen down to −25 °C, after which the device chamber is evacuated, and sublimation removal of moisture takes place.

The dried mixtures were ground using a WF-20B paddle mill (Yueyuehong, China) for 1 or 3 min at a blade speed of 25,000 rpm.

The CNTs/GNPs filler (at a 1:4 component mass ratio) was obtained through ultrasonic treatment of the mixture followed by lyophilic drying and grinding, as in the case of the CNTs/GO particles.

### 2.3. Preparing Epoxy Composites

A hybrid filler amount (0.1–1.0 wt.%) was added to the ED-22 ER, the resulting mixture was stirred by hand and incubated at 20 °C for 1 day. Then, the resulting dispersion was subjected to mechanical processing using an EXAKT 80E three-roller mill (EXAKT Advanced Technologies GmbH, Norderstedt, Germany) at 120 rpm. The gaps between the rolls in the first, second and third runs were 60 and 30 μm, 30 and 15 μm, 15 and 5 μm, respectively. The result was a uniform hybrid filler dispersion in the epoxy resin, to which the UP 606/2 hardener (2 g of the hardener per 100 g of the ER) was added. After stirring for 10 min at 450 rpm, the mixture was vacuumed, poured into molds, incubated at 80 °C for 3.5 h, and then cooled down. After that, the samples were removed from the molds and incubated again at 90 °C for 3.5 h.

Table 1 demonstrates the list of polymer composite samples obtained, their designations, and synthesis conditions.

### 2.4. Material Characterization

The morphology and structure of the samples were studied by using a JSM-6390 LA scanning electron microscope and a JEM 2100F/Cs transmission electron microscope (both—JEOL Ltd, Tokyo, Japan). For SEM investigation, the samples were placed on a conductive carbon tape, then pumped and studied at an acceleration voltage of 20 kV. For TEM analysis, the ground samples from methanol suspensions were placed on a polymer-coated TEM copper grid. The analysis was performed at an accelerating voltage of 200 kV.

The particle sizes of the dispersed phase in the CNTs/GO and CNTs/GNPs hybrid aqueous suspensions were determined by dynamic light scattering using a 308 ZLS analyzer (NICOMP, Urbana, IL, USA).

Raman spectra of the hybrid particle samples were obtained on a DXR Raman Microscope (Thermo Fisher Scientific, Waltham, MA, USA) at an excitation laser wavelength of 532 nm.

Images of the hybrid particles agglomerates in the epoxy resin were obtained using a Micromed-1 optical microscope (Scientific Instruments Co, St. Petersburg, Russia).

Thermogravimetry (TG) curves were constructed for the epoxy composite samples in an air atmosphere using an STA 449 F3 Jupiter synchronous thermal analysis instrument (NETZSCH-Feinmahltechnik GmbH, Selb, Germany). The temperature program included incubation at 30 °C for 10 min and heating from 30 to 900 °C at 10 °C/min.

The electrical resistivity of the epoxy nanocomposites was measured using an E6-13A teraohmmeter (Punane RET, Tallinn, Estonia). The conductivity (σ, S/m) was calculated using the following equation:

(1) $$\sigma =4h/\pi d^{2}R$$

where *h* and *d* are the geometric parameters of the studied sample (height and diameter, respectively), m; *R* is the electrical resistivity, Ohm.

The mechanical properties of the composites were investigated by using an M350-5AT testing machine (Testometric Co, Rochdale, UK) at 50 mm/min (tensile strength) and 20 mm/min (flexural strength).

## 3. Results and Discussion

### 3.1. Studying the CNTs/GO and CNTs/GNPs Hybrid Material Formation

**Figure 1:**
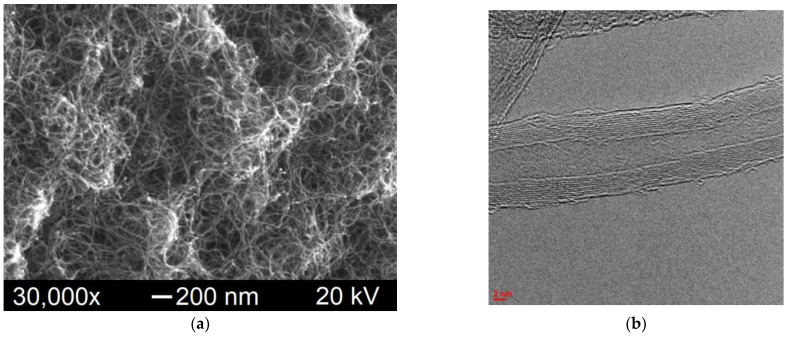

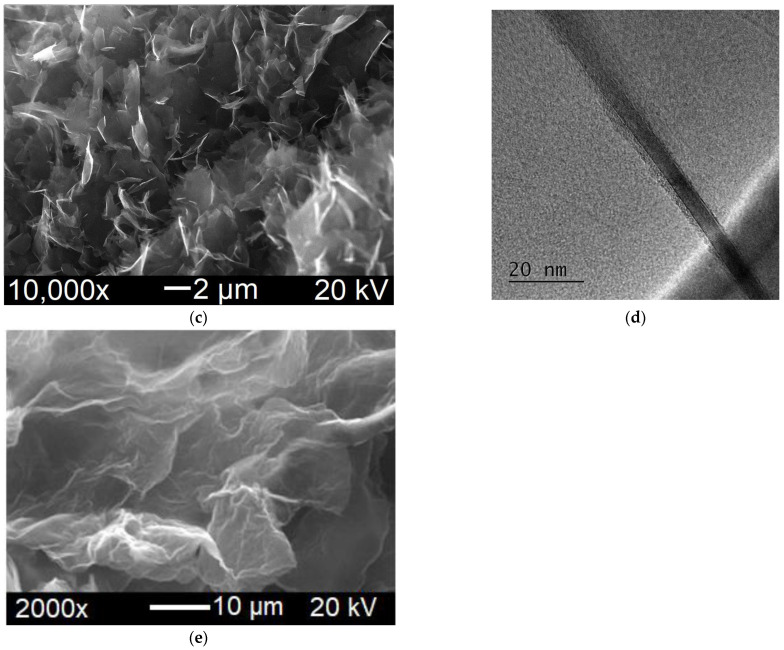
SEM (**a**,**c**,**e**) and TEM (**b**,**d**) images of the original CNTs (**a**,**b**), GNPs (**c**,**d**) and GO (**e**) used.

The electronic images of the original CNMs herein used are shown in Figure 1. The outer diameter of the CNTs varies in the range of 15–30 nm, inner channel diameter is 4–8 nm, length is >2 μm, and the number of graphene layers is 8–20 (Figure 1a,b). The GNPs (Figure 1c,d) are 6–8 nm thick and consist of 15–25 graphene layers. The size of the platelets in the plane varies in the range of 2–10 μm. Unlike the GNPs, the GO (Figure 1e) consists of 1–2 layers. Single graphene nanoplatelets possess a flat surface and sharp edges. The surface of the graphene oxide sheets has a wavy shape.

**Figure 2:**
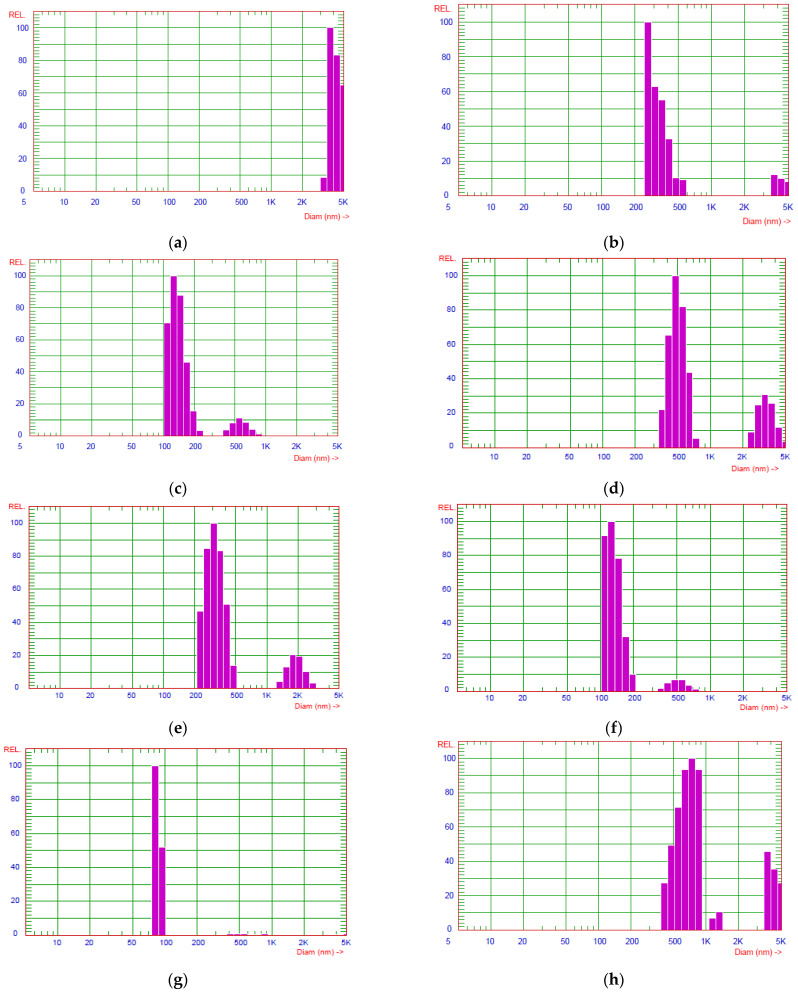
Diagrams of the dispersed phase particle size distribution in the aqueous suspensions containing the original CNTs (**a**), GO (**b**) and GNPs (**c**), as well as the CNTs/GO mixture at the mass ratios of 1:1 (**d**), 1:2 (**e**), 1:3 (**f**) and 1:4 (**g**), and the CNTs/GNPs mixture at the mass ratio of 1:4 (**h**).

At the initial step of the study, the diagram of the dispersed phase particle size (diameter) distribution in the aqueous suspensions containing the initial CNMs, CNTs/GO and CNTs/GNPs mixtures were analyzed (Figure 2). The largest agglomerates greater than 3.5 μm were observed in the initial CNT suspension (Figure 2a), since it is well known that unfunctionalized nanotubes are indeed prone to aggregation in polar solvents.

In the GO (Figure 2b) and GNPs (Figure 2c) suspensions, two fractions of the dispersed phase particles are available. In the case of the GO, their average effective sizes are 300 nm and 4.3 μm. In the GNP suspension, they are somewhat lower, being 140 and 700 nm. This correlates well with the SEM images of the original graphene materials, where one can see that the cross-sectional dimensions of the GO sheets are much larger than those of the single GNP flakes.

For the suspensions where the CNTs:GO ratio was 1:1 (Figure 2d), 1:2 (Figure 2e) and 1:3 (Figure 2f), two scattered radiation peaks, corresponding to the two particle size fractions, can be observed in each diagram. The increase in the GO concentration contributes to the reduction of the particle effective sizes. According to the calculation data, the average particle sizes of the two fractions at the 1:1 CNTs:GO ratio are 496 and 3291 nm. When this ratio is increased to 1:2, the average particle sizes of those fractions decrease to 312 and 1887 nm, and in the case of the 1:3 ratio, they decrease to 131 and 500 nm. Besides, when moving from Figure 2d to Figure 2f, one can observe a trend towards an increase in the relative content of small agglomerates compared to larger ones.

For the suspension with the CNTs:GO ratio of 1:4 (Figure 2g), only a single fraction of the particles, the effective sizes of which vary in a narrow range of 75–100 nm, is available. It should be noted that a further increase in the GO content in the mixture does not significantly change the pattern, i.e., at the 1:4 ratio, it is possible to obtain homogeneous hybrid particles with minimum size.

Thus, the data obtained in the present work confirm the assumption made by the authors of the work [32] that GO acts as a CNT dispersion stabilizer. Moreover, one of our previous works [33] shows, based on molecular dynamics calculations, that GO sheets are arranged around CNTs. When twisting around the CNTs, the transverse GO particles begin to shrink. In this case, the functional groups contained in the GO should prevent the agglomeration of the hybrid particles in polar solvents and polymer matrices.

A different pattern is observed for the CNTs/GNPs suspension (1:4) (Figure 2h). histogram of dispersed phase particle size distribution is almost the same as in the original GNP suspension. This may be due to less flexibility of the nanoplatelets. In this case, one should expect that the CNTs are uniformly distributed on the GNP surface, as a result of which the dispersed phase particle effective size does not change.

**Figure 3:**
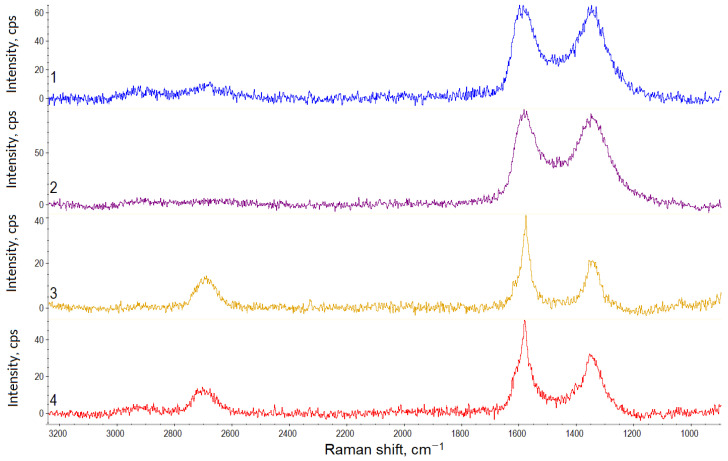
Raman spectra of the hybrid materials: 1—CNTs/GO (1:1); 2—CNTs/GO (1:4); 3—CNTs/GNPs (1:1); and 4—CNTs/GNPs (1:4).

The Raman spectra recorded for the hybrid materials of different compositions after suspension lyophilic drying are shown in Figure 3. The most intense peaks are *D* (1350 cm^−1^) and *G* (1570 cm^−1^), which are typical of carbon nanostructures and caused by the in-plane sp^2^ and sp^3^ vibrations of carbon atoms, respectively [34]. The integrated intensity ratio, *D*/*G*, is widely used to characterize the number of defects in carbon materials [35].

The *2D* peak (2700 cm^−1^) is sensitive to the *π*-band in the electronic structure. The changes in its position and intensity are related to the availability and localization of oxygen-containing groups in the basal plane [36].

According to [36,37,38,39], the small peaks—*D*′ (~1620 cm^−1^), *D*′’(~1500 cm^−1^), and D* (~1150 cm^−1^)—also characterize the availability of defects and the oxygen-containing groups. The *D*+*G* peak (2920 cm^−1^) is detected in the spectra of materials having highly disordered structures [36].

The results of the Raman spectra processing are presented in Table 2.The hybrid materials demonstrate different characteristics depending on the graphene component type and amount. The *G* peak on the CNTs/GNPs Raman spectra is shifted to the region of higher wavenumbers. Besides, the 2*D*/*G* ratio for the CNTs/GNPs is higher than for the CNTs/GO. Both ratios indicate a smaller number of graphite layers in the CNTs/GNPs hybrid particles. This is a paradoxical fact, given that the GNPs contain significantly more layers than the GO. Obviously, when the hybrid material is formed, in the case of using the GO, more intense aggregation occurs during the material drying than in the case of using the GNPs. The different nature of the effects of GO and GNPs on the self-assembly process is indicated by the fact that with an increase in the GO content, the 2*D*/*G* value decreases, whereas an increase in the GNP content has the opposite effect.

Higher defect ratio (*D*/*G*) values for the CNTs/GO compared to the CNTs/GNPs may be due to the higher GO oxidation. Besides, the CNTs possess a higher defect ratio index in comparison with the GNPs, but a lower one if compared to the GO. The different nature of the *D*/*G* changes with increasing CNTs contents may be associated with differences in the CNTs’ location relative to the graphene component. The nanotubes can easily be located between the GO layers in the CNTs/GO material, whereas in the CNTs/GNPs, the nanotubes will be located at the nanoplatelets surface or edges.

The *D*′/*G* and *D*/*G* values correlate with each other. At the same time, there is a significant difference in the D’/G values for the CNT/GO depending on the component ratio. Considering that particles of the same type are found in the suspensions at the CNTs:GO ratio of 1:4, it can be assumed that in this case, the entire surface of all the nanotubes is covered with the GO layers.

For planar carbon nanostructures such as graphene, the *D*/*D*′ ratio value provides information about the predominant types of defects on the surface [40]. It is noticeable that the hybrid materials containing the minimum amount of CNTs demonstrate similar values. In this case, flat graphene layer edges represent the predominant type of defect. As the CNTs’ concentration in the CNTs/GO increases, the *D*/*D*′ value decreases down to the values which are usually characteristic of the predominant local surface graphene layers defects. For the CNTs/GNPs materials, the *D*/*D*′ value increases with increasing CNT concentration. This may be due to the fact that the CNTs form large agglomerates on the graphene platelets surface, and the end sections of the nanotubes are recognized as additional edge defects.

**Figure 4:**
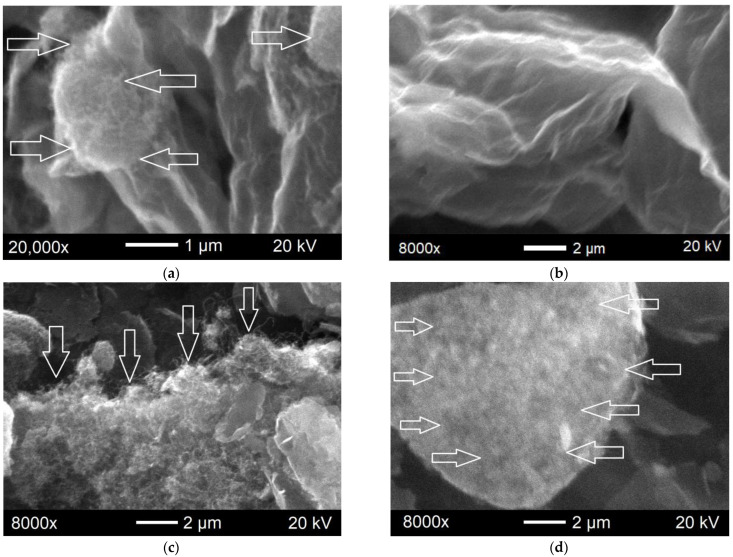
SEM images of the hybrid materials: CNTs/GO (1:1) (**a**); CNTs/GO (1:4) (**b**); CNTs/GNPs (1:1) (**c**); and CNTs/GNPs (1:4) (**d**). The arrows show CNT localization sites.

The assumptions made in the Raman spectra analysis are consistent with the data on the morphology of the hybrid materials obtained through electron microscopy (Figure 4).

The SEM image of the CNTs/GO (1:1) (Figure 4a) shows loose CNT agglomerates of round shape with the size of 2–2.5 μm located on the GO sheets surface. Given that this material has a fairly high CNT content, one can assume that some of the nanotubes are already located between the GO layers.

In the SEM image of the CNTs/GO (1:4) (Figure 4b), the nanotubes are not detected, since they are all covered by the GO layers. The graphene component surface becomes more curved as, apparently, the GO sheets are indeed located over the nanotube agglomerates surface, tending to take their shape. The main type of structure defects in the hybrid material in this case are surface defects of the graphene layers, as indicated by the Raman spectroscopy data.

The formation of the hybrid material from the CNTs and GNPs occurs in a quite different manner. The CNTs almost completely cover the surface of the remaining straight graphene scales (Figure 4c,d). In the material with a higher CNTs content, the nanotube layer on the nanoplatelets surface looks non-uniform in thickness (Figure 4c). In the CNTs/GNPs mixture (1:4), the nanotubes are more uniformly distributed on the nanoplatelet surface (Figure 4d). Part of the nanoplate remains uncovered. These data are in complete agreement with the results of measurements of the dispersed phase particle effective sizes and the assumptions made in the Raman spectra analysis.

**Figure 5:**
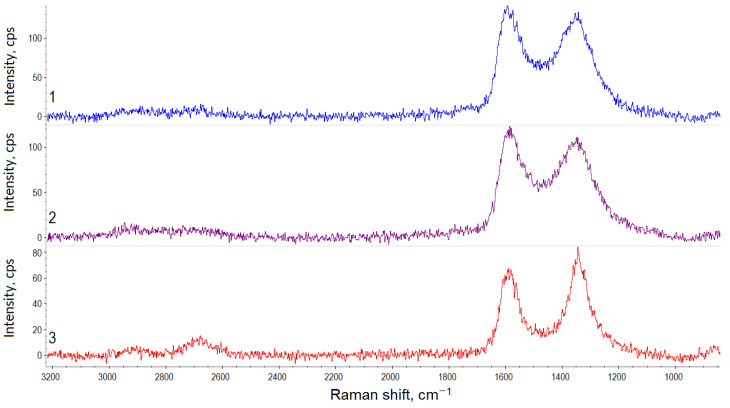
Raman spectra of the CNTs/GO hybrid material (1:1) after lyophilic drying (1), lyophilic drying and mechanical grinding (2), drying at 40 °C, and mechanical grinding (3).

A certain influence on the structure and defectiveness of the hybrid particles is exerted by the method of drying and grinding the material (Figure 5). Grinding the hybrid material in a paddle mill increases the relative intensity of the *D* peak on the Raman spectra. The mechanically ground material after drying at 40 °C has a less defective structure. A more detailed analysis of the Raman spectroscopy data (Table 3) confirms the initial conclusions.

It should be noted that when dried at 40 °C, the hybrid material forms very strong agglomerates that are difficult to grind. Apparently, due to this, even after the grinding, the material is characterized by a relatively low *D*/*G* value. The lyophilic drying process results in a porous material having many defects. Its mechanical grinding contributes to an increase in the *D*/*G* value due to the appearance of additional fracture surfaces of the hybrid particles. The *D*/*G* and *D*′/*G* value changes correlate with each other. However, the *D*”/*G* value decreases during the mechanical processing, indicating the formation of relatively homogeneous particles with a higher degree of crystallinity than in the native dried material.

All the samples demonstrated relatively low *D*/*D*′ values, which is usually typical of materials that are dominated by defects on the graphene layers surface.

### 3.2. Modifying the Epoxy Resin with the CNTs/GO and CNTs/GNPs Hybrid Fillers

As stated in [41], using a three-roll mill is the most efficient way to disperse CNMs in an ER. However, in addition to the mixing mode, the composition of filler particles and their concentration affect the distribution character.

**Figure 6:**
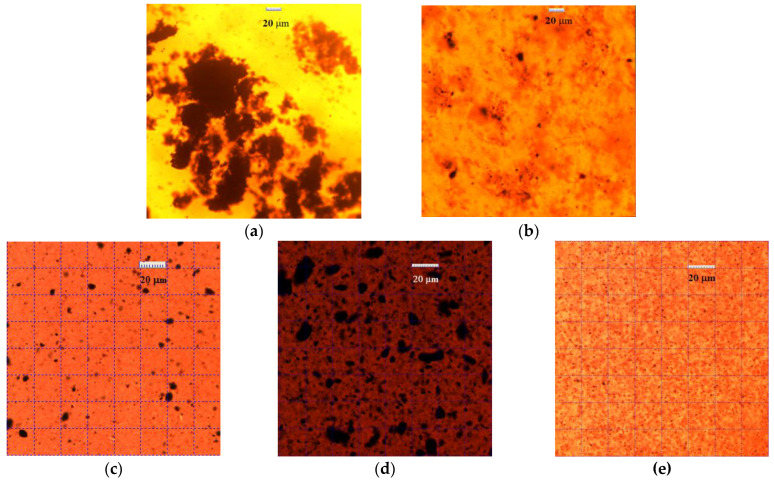
Microphotographs of the 0.20%CNT:GNP(1:1)F (**a**), 0.20%CNT:GNP(1:4)F (**b**) 0.20%CNT:GO(1:1)F* (**c**), 1.00%CNT:GO(1:1)F* (**d**) and 0.20%CNT:GO(1:4)F* (**e**) suspensions in the ED-22 ER after processing in the EXAKT three roller mill.

Figure 6a,b present the microphotographs of the CNTs/GNPs hybrid filler suspensions at the 1:1 and 1:4 component ratios. In the first case (Figure 6a), large loose agglomerates of irregular shape with sizes up to several hundred μm can be detected. According to the previously reported SEM data, in this filler, the CNTs cover the GNP surface with a massive irregular layer, in which the presence of CNT agglomerates not connected with the graphene component surface, is not excluded. The GNPs flakes containing polar functional groups practically do not contact with the ER, as a result of which they cannot ensure uniform distribution of the hybrid filler in the polymer matrix.

With higher GNPs contents, the image background appears darker, due to a more uniform distribution of the hybrid carbon filler (Figure 6b). Numerous agglomerates, heterogeneous in shape and size, the diameters of which do not exceed 20–30 μm, are found in the suspension composition. In this case, a part of the GNP surface remains free of the CNTs and provides interaction of the hybrid filler with the ER.

The epoxy suspensions containing the CNTs/GO hybrid fillers look different (Figure 6c,d). In the 0.20 wt.% CNTs/GO (1:1) dispersion, there are numerous agglomerates of predominantly round shape up to 1 μm in size, but there are also few dense agglomerates up to 10 μm in size (Figure 6c). An increase in the CNTs/GO (1:1) filler concentration by a factor of 5 promotes aggregation of the particles in the dispersed phase. In the field of view, quite a lot of agglomerates with sizes of 20–30 µm (Figure 6d) are available. The CNTs/GO (1:4) filler forms the most uniform dispersion in the epoxy resin with agglomerate sizes (diameters) no larger than 0.5 μm (Figure 6e). Thus, it can be assumed that the hybrid material of this composition will disperse equally well in polar solvents and polymer matrices.

### 3.3. Studying the Characteristics of the Hardened Epoxy Nanocomposites

#### 3.3.1. Electrical Conductivity

According to the data presented in Table 4, all the prepared epoxy nanocomposites practically do not differ in the conductivity properties from the native polymer matrix and represent dielectrics. This is an expected result considering that in this study, oxidized graphene materials, characterized by low conductivity, were used to stabilize the dispersions [42]. The GO and GNPs interfere with the contact of the CNTs with each other, which does not contribute to the implementation of the conductive capabilities of the nanotubes.

Furthermore, numerous works indicate that the mass fraction of nanocarbon fillers in the epoxy composite should be at least 1 wt.% to form a percolation circuit and to ensure high conductivity values [43].

According to [44], the high proportion of CNT and graphene loadings contributes to the reduction of the mechanical properties of polymer composites. In this study, the authors did not introduce more than 1 wt.% of the hybrid fillers into the ER to avoid deterioration of the composite strength characteristics. However, it can be stated that the materials, the composition of which is shown in Table 4, can be used in applications where electrical insulation is required.

In some cases (e.g., for the 0.25%CNT:GO(1:1)T and 1.00%CNT:GO(1:4)F samples), the electrical conductivity of the composites is even slightly lower than that of the unmodified ER. The reason for this result cannot be explained exactly. Apparently, in these cases, the polymer structure is rearranged, thereby leading to an increase in its electrical resistivity. A similar effect is demonstrated in [45] using the example of epoxy composites filled with carbon-based materials.

#### 3.3.2. Thermogravimetric Analysis

**Figure 7:**
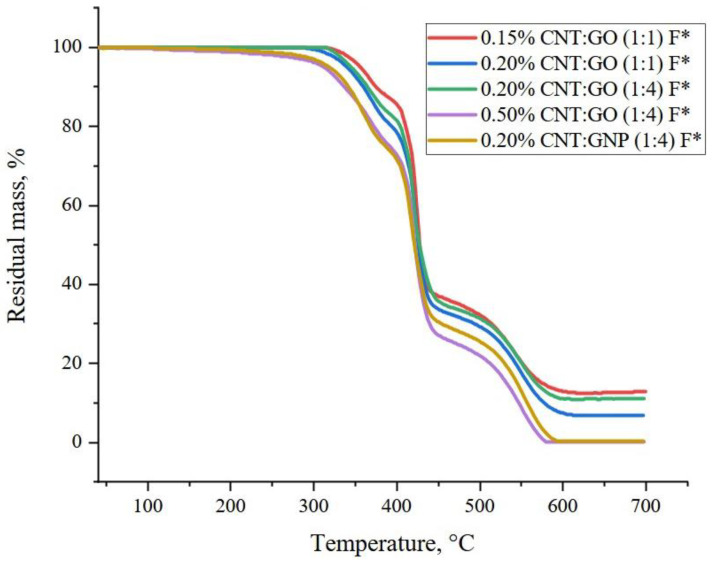
TG curves of the epoxy nanocomposite samples (the “*” sign means that they were ground for an increased period of time).

Thermogravimetric analysis (TGA) is a way to evaluate the thermal stability of materials. All the TGA curves of the composites presented in Figure 7 have a similar character. Two stepwise sections of mass decrease in the temperature ranges of 250–430 °C and 430–600 °C can be distinguished on them. The samples containing 0.50 wt.% CNTs/GO (1:4) and 0.20 wt.% CNTs/GNPs (1:4) start undergoing thermal degradation earlier than the others, and eventually lose up to 100% of their mass. The composites containing 0.20 wt.% CNTs/GO (1:1) and CNTs/GO (1:4) are more stable. Their TG curves are close to each other. The composite with the lowest content (0.15 wt.%) of the CNTs/GO (1:1) is the most thermally stable. The latter three materials are thermally stable in air at temperatures up to 300 °C.

In general, one can note a trend towards a decrease in resistance to the thermal degradation with an increase in the hybrid filler content. This can be explained by the fact that interacting with the epoxy resin, the carbon nanostructures reduce the degree of polymerization upon hardening, which leads to some decrease in the thermal stability.

Thus, owing to the modification with the hybrid fillers, the temperature range for the operation of the epoxy composites can be extended.

#### 3.3.3. Mechanical Properties

**Figure 8:**
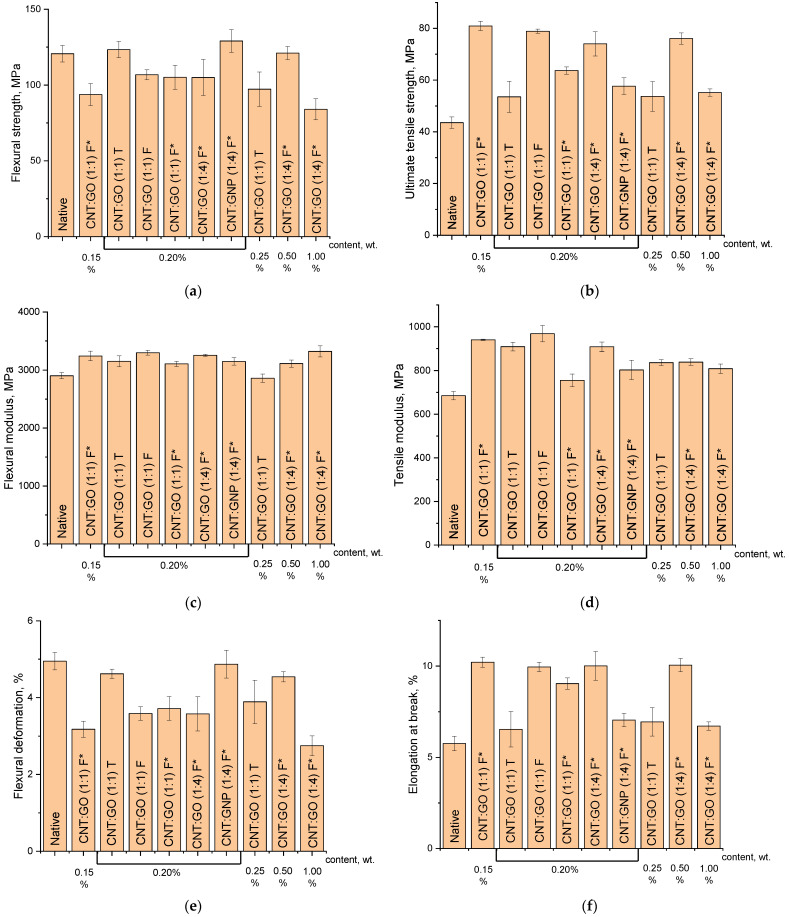
Flexural (**a**,**c**,**e**) and tensile (**b**,**d**,**f**) characteristics of the epoxy samples (the “*” sign means that they were ground for an increased period of time).

The introduction of the hybrid fillers affects the properties of the epoxy composites under different types of mechanical action. The left side of Figure 8 shows the changes in the flexural strength (Figure 8a), flexural modulus (Figure 8c) and relative strain (Figure 8e) of the unmodified material and epoxy nanocomposites containing different hybrid filler options.

Most of the composite samples possess lower flexural strength than the native material does. In this parameter, only the 0.20%CNT/GO(1:1)T and 0.20%CNT/CNP(1:4)F* samples are 2 and 8% better, respectively, than the native sample. These composite samples are characterized by approximately the same relative flexural deformation values as the unmodified material. At the same time, the flexural modulus of almost all the composites is 7–14% better than that of the native sample.

Thus, the hybrid materials having similar 2*D*/*G* ratio values on the Raman spectra proved to be the best as modifiers here (Table 2). It should be reminded that this indicator is related to the number of graphene layers in the hybrid particles, and, consequently, to the nature of their aggregation. However, in addition to the composition of the filler, its concentration in the composite is also important.

In the case of the CNT/GO(1:1)T sample, increasing the concentration from 0.20 to 0.25% leads to a sharp decrease in the flexural strength of the epoxy composite, which may be associated with an increase in the filler particle size in the matrix and the appearance of additional deformation sites for this reason.

It should be noted that the 0.20%CNT:GO(1:4)F* sample, with the finest dispersed particles (Figure 6e), did not show high results. Consequently, the uniformity of the filler dispersion in the polymer matrix does not always provide the maximum strength characteristics of the composite.

The hybrid fillers possess a much greater positive effect on the tensile mechanical properties of the composite (the right side of Figure 8). All the composites have an ultimate tensile strength value higher than that of the unmodified material (Figure 8b). In this parameter, the 0.15%CNT:GO(1:1)F*, 0.20%CNT:GO(1:1)F* and 0.50%CNT:GO(1:4)F* samples appear to be 88, 84 and 77%, respectively, better than the native one. These samples also show the highest elongation at break. It increases from 5.8% for the native material to 10.21 and 10.1% for the 0.15%CNT:GO(1:1)F* and 0.50%CNT:GO(1:4)F* samples, respectively (Figure 8f). An increase in the both tensile strength and elongation at break has previously been observed in CNM-modified epoxy composites [46]. The authors of the work [47] explain this by the GO positive effect on the segmental mobility.

The tensile modulus of all the composites containing the hybrid fillers was found to be higher than that of the unmodified epoxy resin. This parameter is ~40% higher for the 0.20%CNT:GO(1:1)F* sample than for the native one (Figure 8d).

The analysis of the obtained data showed that the maximum values of the tensile strength characteristics are not always demonstrated by the epoxy composites prepared using the CNTs/GO (1:4) mixture. The best performance is shown by the CNTs/GO (1:1) mixtures, in which, according to Figure 4a, part of the nanotubes is not covered with the graphene sheets and can further provide a reinforcing effect, whereas the GO helps to maintain the mobility of the polymer matrix segments.

#### 3.3.4. SEM Data on the Composite Fracture

According to [48], the fracture of an ER is accompanied by the localization of stresses on its surface or inside its volume. Under the action of a mechanical load, the formation and growth of cracks occurs. The introduction of hybrid fillers contributes to a change in the mechanism of epoxy composite fracture.

**Figure 9:**
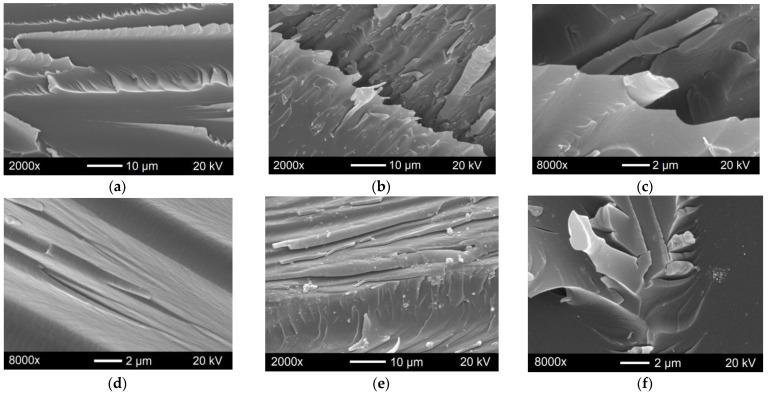
SEM images of the native ER (**a**) and 0.20%CNT:CNP(1:4)F epoxy composite (**b**,**c**) surface fractured under flexural loading, and the native ER (**d**) and 0.15%CNT:GO(1:1)F* epoxy composite (**e**,**f**) surface fractured under tensile break.

In the SEM images, the surface of the native epoxy material appears relatively smooth after bending (Figure 9a) and stretching (Figure 9d). It does not have sharp protrusions and recesses. This indicates the brittle nature of the material fracture.

The SEM images of the fracture surface of the 0.20%CNT:GNP(1:4)F* composite under flexural loading show deep cracks with rough edges, thereby indicating a strong interaction of the polymer matrix with the hybrid filler which has a layered structure (Figure 9b,c).

The 0.15%CNT:GO(1:1)F* composite demonstrated the maximum tensile strength and tensile modulus. The SEM image of the surface of this material (Figure 9e,f) reveals fragments indicating a plastic fracture mechanism. In some areas, one can observe sharp roughness, the formation of which is possible in sites of accumulation of nanotubes not covered with the GO sheets.

Thus, the composite samples showing the best mechanical properties differ greatly in the fracture mechanism from the native ER. The properly prepared CNTs/GNPs filler of optimal composition contributes to some strengthening of the composite under bending loads.

The introduction of the CNTs/GO filler changes the fracture mechanism of the epoxy composite from brittle to ductile, which is reflected in the mechanical characteristics of the material under tension.

## 4. Conclusions

Two types of graphene materials (GO and GNPs), differing in the number of graphene layers and planar size, were employed herein to prepare mixtures with cylindrical CNTs, which then were used as hybrid modifiers of the ER.

It was shown that increasing the graphene component concentration contributes to the stabilization of the CNT dispersions in water and the ER. The nanotubes were found to be located between the GO sheets in the CNTs/GO mixture and on the GNP surface in the CNTs/GNPs mixture.

The final properties of the hybrid fillers are affected by the way of drying and grinding of the materials before introduction into the ER. The best performances are characterized by the hybrid materials after lyophilic drying and minimum duration of the mechanical grinding.

In the ER, the CNTs/GO hybrid filler is dispersed more uniformly than the CNTs/GNPs is. The size of the CNTs/GO (1:4) agglomerates does not exceed 500 nm.

It was found that the uniform distribution of the hybrid filler in the epoxy matrix does not contribute to the maximum strength characteristics of the composite. The nanocomposites containing 0.15–0.20 wt.% CNTs/GO mixtures in a 1:1 mass ratio contribute to 84–88 and 40% increases in the tensile strength and elastic (Young’s) modulus, respectively. At the same time, the elongation at break also increases.

Regardless of the nature of the graphene component used in the hybrid filler, all the epoxy composites obtained are dielectric and resistant to heating in the air up to 300 °C.

Due to their performance values, the CNTs/GO and CNTs/GNPs hybrid fillers-based epoxy composites can be used as structural materials in various fields of engineering.

## Figures and Tables

**Table 1:** Epoxy composite samples, their designations, and synthesis conditions (the “*” sign means that they were ground for an increased period of time).

Sample Designation	Hybrid Filler Composition (Component Mass Ratio)	Drying Condition	Grinding Time, min	Hybrid Filler Mass Content in Composite, %
Native	-	-	-	0
0.15%CNT:GO(1:1)F*	CNTs and GO (1:1)	lyophilic	3	0.15
0.20%CNT:GO(1:1)T	CNTs and GO (1:1)	at 40 °C	1	0.20
0.20%CNT:GO(1:1)F	CNTs and GO (1:1)	lyophilic	1	0.20
0.20%CNT:GO(1:1)F*	CNTs and GO (1:1)	lyophilic	3	0.20
0.20%CNT:GO(1:4)F*	CNTs and GO (1:4)	lyophilic	3	0.20
0.20%CNT:GNP(1:4)F*	CNTs and GNPs (1:4)	lyophilic	3	0.20
0.25%CNT:GO(1:1)T	CNTs and GO (1:1)	at 40 °C	1	0.25
0.50%CNT:GO(1:4)F*	CNTs and GO (1:4)	lyophilic	3	0.50
1.00%CNT:GO(1:4)F*	CNTs and GO (1:4)	lyophilic	3	1.00

**Table 2:** Raman spectroscopy data on the CNTs/GO and CNTs/GNPs hybrid materials obtained after lyophilic drying of the aqueous suspensions.

Characteristics		Hybrid Material Composition			
		CNTs/GO (1:1)	CNTs/GO (1:4)	CNTs/GNPs (1:1)	CNTs/GNPs (1:4)
Peak *G* position, cm^−1^		1575	1577	1580	1583
Integrated intensity ratio	*D*/*G*	1.395	1.217	0.541	0.617
	*D*′’/*G*	0.372	0.290	0.081	0.085
	*D*′/*G*	0.744	0.362	0.108	0.170
	*D*/*D*′	1.875	3.360	5.000	3.625
	*2D*/*G*	0.256	0.087	0.378	0.298

**Table 3:** The Raman spectra analysis for the CNTs/GO (1:1) hybrid materials obtained in different drying and grinding modes.

Drying Condition	Mechanical Grinding	Intensity Ratio			
		*D*/*G*	*D″/G*	*D*′/*G*	*D*/*D*′
lyophilic	−	1.312	0.269	0.699	1.877
lyophilic	+	2.020	0.157	1.588	1.272
at 40 °C	+	1.795	0.308	0.821	2.188

**Table 4:** The electrical conductivity of the epoxy composite samples (the “*” sign means that they were ground for an increased period of time).

Sample	Electrical Conductivity, S/m
Native	1.08 × 10^−11^
0.15%CNT:GO(1:1)F*	1.57 × 10^−11^
0.20%CNT:GO(1:1)F	1.05 × 10^−11^
0.25%CNT:GO(1:1)T	8.91 × 10^−12^
0.20%CNT:GO(1:1)T	2.07 × 10^−11^
0.20%CNT:GO(1:1)F*	1.38 × 10^−11^
0.20%CNT:GO(1:4)F*	1.31 × 10^−11^
0.50%CNT:GO(1:4)F*	1.05 × 10^−11^
1.00%CNT:GO(1:4)F*	1.03 × 10^−12^
0.20%CNT:GNP(1:4)F*	1.16 × 10^−11^

## Data Availability

The data supporting the reported results were generated during the study.

## Abbreviations

carbon nanomaterials	CNMs
carbon nanotubes	CNTs
multiwalled carbon nanotubes	MWCNTs
carbon nanofibers	CNFs
graphene nanoplatelets	GNPs
graphene oxide	GO
epoxy resin	ER
